# Development of a Method for the Detection, Quantification, and Validation of 4‐Oxo‐2‐Nonenal in Cooked Meat

**DOI:** 10.1155/ianc/6342679

**Published:** 2025-12-11

**Authors:** Fidele Benimana, Anupam Roy, Anand Mohan

**Affiliations:** ^1^ Department of Food Science and Technology, University of Georgia, 100 Cedar Street, Athens, Georgia, 30602, USA, uga.edu; ^2^ Laboratory of Applied Food Chemistry, Technology and Process Engineering, Department of Chemical Engineering, Birla Institute of Technology, Ranchi, Jharkhand, 835215, India, bitmesra.ac.in

**Keywords:** 4-oxo-2-nonenal, LOD, LOQ, precision, robustness, sensitivity, ultrahigh performance liquid chromatography

## Abstract

This research mainly focused on developing and validating the ultraperformance liquid chromatography photodiode array (UPLC‐PDA) method to analyze 4‐oxo‐2‐nonenal (4‐ONE) in fully cooked meat products. The UPLC‐PDA method developed has advantages such as sensitivity, excellent resolution, and fast separation through chromatography. To ensure its reliability and accuracy, the method underwent validation processes for linearity, precision, accuracy, robustness, and limit of detection (LOD) and quantification (LOQ). The results demonstrated a linear relationship between concentration and response within the 0.0032–10 ng/mL range with a correlation coefficient of *R* ^2^ ≥ 0.9993. The method also exhibited precision with relative standard deviations (RSDs) below 2% for both intraday and interday analyses. Moreover, recovery studies confirmed the method’s accuracy, with percent recoveries ranging from 97.16% to 105.9%. Furthermore, the results for LOD and LOQ were 0.03 and 0.091 ng/mL, respectively. Lastly, it was concluded that the developed method remains reliable under certain conditions by varying parameters such as the flow rate, mobile phase composition, and detection wavelength in robustness evaluations. This developed UPLC‐PDA technique offers a reliable and effective means of identifying and measuring 4‐ONE in cooked meat. It plays a role in ensuring food safety and addressing health issues associated with its consumption.

## 1. Introduction

Meat consumption has steadily increased due to its significance as a key source of protein and fat in the human diet. Global meat consumption is expected to increase by 14% by 2030 [1]. Among its many attributes, texture and flavor are two of the most appealing qualities of meat, which are primarily influenced by its chemical composition and cooking methods. However, cooking methods such as frying cause undesirable chemical changes, particularly lipid oxidation, which produces harmful compounds that can affect sensory quality and safety [2, 3].

**Figure 1:**
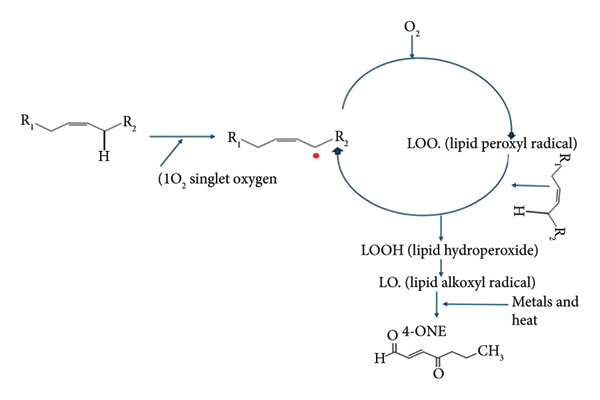
General schematic representation of lipid oxidation pathways leading to the formation of lipid oxidation byproducts including 4‐oxo‐2‐nonenal (4‐ONE) in food systems.

Polyunsaturated fatty acids (PUFAs), which are abundant in meat lipids, are highly susceptible to oxidation when exposed to heat, oxygen, and moisture during cooking [4, 5]. During lipid peroxidation, reactive oxygen species (ROS), such as hydroxyl radicals (OH) and singlet oxygen (.1O_2_), attack unsaturated fatty acids’ double bonds, leading to the formation of lipid hydroperoxides, which are unstable and decompose into lipid oxidation secondary end products such as aldehydes, ketones, and other volatile compounds (Figure 1) [6, 7]. Among secondary end products generated, there is 4‐oxo‐2‐nonenal (4‐ONE; C_9_H_14_O_2_), an α,β‐unsaturated aldehyde bearing a 4‐keto substituent that confers marked electrophilicity. In cooked meat systems, 4‐ONE predominantly arises from the oxidation of ω‐6 PUFAs, such as linoleic acid, via the fragmentation of 9‐ and 13‐hydroperoxyoctadecadienoic acid (HPODE) intermediates, followed by cyclization/rearrangement [8, 9]. Based on its strong Michael‐acceptor character, 4‐ONE readily forms adducts and crosslinks with nucleophilic amino acid side chains (e.g., Cys, His, and Lys), lipids, and nucleic acids. Such reactions not only drive sensory deterioration but have been implicated in oxidative stress, chronic inflammation, and related pathophysiology [10–14]. Consequently, sensitive and practical quantification of 4‐ONE in cooked meats is essential for risk assessment, process control, and mitigation strategies.

Several analytical methods have been reported for 4‐ONE detection and measurement, including GC‐MS [15], LC‐MS/MS [16], and HPLC‐MS/MS [17] with UV/fluorescence detection (often after derivatization). While mass spectrometry techniques provide excellent sensitivity and structural information, they can be limited in routine quality control and quality assurance due to the need for labor‐intensive derivatization, longer analysis times, higher costs, and limited instrument availability. In contrast, ultraperformance liquid chromatography with photodiode‐array detection (UPLC‐PDA) offers advantages in analyte detection and measurement while being more cost‐effective and faster.

**Figure 2:**
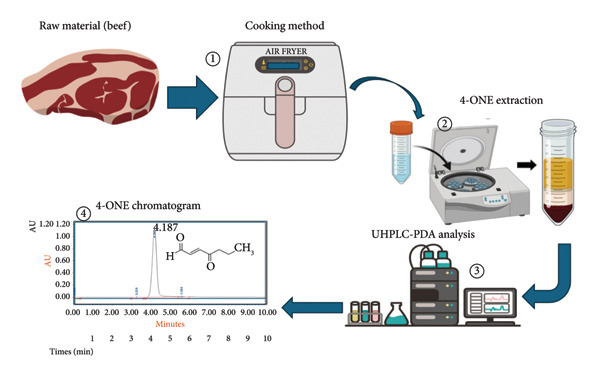
A diagrammatic representation of the methodology workflow.

To our knowledge, no UPLC‐PDA method has been reported for directly detecting and quantifying 4‐ONE in food matrices [8, 14, 18]. This work addresses that gap by developing and validating a rapid, cost‐effective UPLC‐PDA method for 4‐ONE in cooked meat (Figure 2). This research focuses on chromatographic optimization on an ACQUITY UPLC BEH C‐18 (1.7 μm) column, spectral selection, and full performance characteristics (linearity, limit of detection [LOD]/limit of quantification [LOQ], precision, accuracy/recovery, robustness, and matrix effects). The method enables routine monitoring of 4‐ONE in industrial and research environments by offering a pragmatic alternative to MS‐intensive workflows.

## 2. Materials and Methods

### 2.1. Instrumentation and Software

The analysis was performed using a Waters Acquity UPLC‐H Class system, equipped with a quaternary solvent system, autosampler, and PDA detector (190–500 nm). Separation was carried out on a Waters Acquity BEH C18 column (150 × 2.1 mm, 1.7 μm). Data acquisition and processing were performed using Empower software. For chromatographic separation, mobile phase A (methanol) and mobile phase B (acetonitrile) were used in a 75:25 (v/v) ratio at a flow rate of 0.03 mL/min. Methanol was also used as the diluent. We injected 1 μL of the sample into the column, and the column temperature was maintained at 25°C using the UPLC system’s built‐in thermoregulation function controlled by Empower software. The eluent was monitored at a wavelength of 221 nm. To ensure the purity of our sample and mobile phases, we employed a Millipore filter assembly equipped with a 0.45‐μm nylon membrane, following a 15‐min sonication, as an added precaution to remove any particulates or contaminants that could be introduced during solvent handling and to protect the UPLC column from blockage or performance issues.

### 2.2. Materials

Acetonitrile (ACN) and methanol were of HPLC grade. Trichloroacetic acid (TCA), butylated hydroxytoluene (BHT), and diethylenetriaminepentaacetic acid (DTPA) were obtained from Sigma‐Aldrich. The 4‐ONE standards were purchased from Cayman Chemical (Ann Arbor, MI), and ethanol and methanol used in this research were of analytical grade.

### 2.3. Preparation of 4‐ONE Stock Solutions

Stock solution was prepared by dissolving 50 mL of 4‐ONE (5 g/mL) in methanol. Then, standard solutions of 10, 0.4, 0.2, 0.08, 0.016, and 0.032 ng/mL were subsequently prepared from the stock solution.

### 2.4. Sample Extraction

We employed the techniques developed by Klein et al. [19] with minor modifications. First, we took 5 g of cooked beef and homogenized it in a mixture of 100 mL of 80% ethanol (containing 10% TCA at a pH level of 3.5). This was performed using an Ultra Turrax homogenizer at a speed of 3000 rpm. Then, aliquots of the samples were dispensed into Eppendorf tubes (1.5 mL) for further analysis. Next, the samples were centrifuged at 10,000 g for 5 min at 4°C. Afterward, a 5‐mL aliquot was withdrawn from the supernatant, and 250 μM of BHT and 500 μM of DTPA were added in methanol. Samples were vortexed for 5 min to ensure thorough mixing. The solutions were then incubated at 45°C for 5 h to allow for the appropriate reactions. Following this incubation period, the solutions were cooled on ice and underwent another round of centrifugation at a force of 10,000 *g* for 10 min at 4°C temperature. This ensured that all proteins present in the solution were effectively precipitated. Only 2 mL of the resulting supernatant were used after being filtered through a syringe filter with a pore size of 0.45 μm for detection and quantification analyses using an instrument coupled with a PDA detector.

### 2.5. Method Validation, Linearity, and Range of Detection

#### 2.5.1. Precision

To examine the precision of the developed method, six injections of five different concentrations were prepared on the same day of the study, and the relative standard deviation (% RSD) was calculated to verify the intra‐day precision. The same procedure was repeated for interday precision on three nonconsecutive days within the same week to evaluate variability across different analytical runs.

#### 2.5.2. Accuracy/Recovery

To evaluate the accuracy, we conducted recovery studies. We added three known concentrations of 4‐ONE to the mixture of extraction solutions. Then, we estimated the recovery of the added 4‐ONE as follows:

(1) Recovery,%=observed concentration of 4−ONEspiked concentration of 4−ONE∗100.

#### 2.5.3. Robustness

To assess the method’s robustness, we investigated the impact of parameter adjustments, including the flow rate, mobile phase, and detection wavelength. During this evaluation, one parameter was modified while keeping the remaining parameters constant.

#### 2.5.4. LOD and LOQ

The UPLC‐PDA method sensitivity evaluation includes the determination of both the detection limit (LOD) and the quantification limit (LOQ). The LOD represents the smallest detectable amount of analyte above background noise, while the LOQ represents the lowest concentration that can be measured with acceptable precision and accuracy [20–22]. The following equations utilized the ICH guidelines to determine these parameters by using the calibration curve slope (S) and response standard deviation (SD).

(2) LOD=3.3∗SDS,LOQ=10∗SDS.

#### 2.5.5. Statistical Analysis

Statistical analyses were performed using R and Microsoft Excel. Data were analyzed using nonparametric one‐way ANOVA, with a significance threshold of *p* > 0.05.

## 3. Results and Discussion

### 3.1. Optimization of UPLC Parameters

#### 3.1.1. Flow Rate

**Figure 3:**
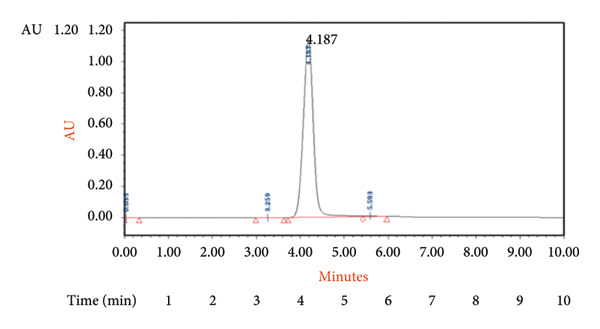
Chromatogram of ultraperformance liquid chromatography (UPLC) generated for 4‐oxo‐2‐nonenal (4‐ONE) at a flow rate of 0.03 mL/min.

The speed at which a sample moves through a column, referred to as the flow rate, plays a role in the separation process. It directly impacts how quickly the mobile phase travels and influences factors such as resolution, peak shape, and overall efficiency of chromatography [23–25]. Lower flow rates can enhance resolution but may extend analysis times. Comprehending and adjusting the flow rate appropriately can achieve separations that advance research and analysis across various scientific domains [26]. In our study, we observed that a flow rate of 0.03 mL/min yielded a sharp peak with excellent resolution compared to other flow rates used​ (Figure 3).

#### 3.1.2. Sample Volume Injection

The amount of sample injected in ultraperformance liquid chromatography (UPLC) impacts the outcomes of the process. When a smaller injection volume is used, the chromatogram peaks appear sharper and narrower [27, 28]. Selecting an injection volume that aligns with the sample concentration and column capacity is crucial for achieving optimal analysis performance. Adjusting the injection volume can enhance the peak resolution, sensitivity, and overall quality of their chromatograms, while ensuring consistent results across various analytical applications [29]. In our study, an injection volume of 1 μL produced a sharp, symmetrical 4‐ONE peak with a retention time (RT) of 4.2 min for the matrix sample and 4.18 min for the standard, confirming consistent identification between the sample and the standard. Under these conditions, replicate injections met the precision target (≤ 2% RSD) established for this assay. Accordingly, 1 μL was selected for all subsequent analyses because it maintained peak shape and resolution while supporting the method’s sensitivity (LOD = 0.03 ng mL^−1^ and LOQ = 0.091 ng mL^−1^).

#### 3.1.3. Mobile Phase

The selection and optimization of the phase play a role in the separation process during chromatographic analysis [30]. In studies concerning secondary oxidation, aldehyde compounds, such as 4‐ONE, have been used in conjunction with mobile solvents like ACN, MeOH, ammonium acetate, and water [31–33]. However, combining ACN and MeOH as organic solvents in the mobile phase offers distinct advantages. Compared with MeOH, ACN has a higher dipole moment, which enhances elution efficiency in reverse‐phase chromatography [34]. On the other hand, using ACN and MeOH helps reduce viscosity compared to using ACN alone. This reduction in viscosity directly impacts the flow rate and analysis duration by alleviating pressure within the column. Consequently, it ensures a separation process within a timeframe. [35]. In our study, we tested the effectiveness of combining methanol and acetonitrile and found that it produced good results compared with using either solvent in our experiment, giving 4‐ONE a RT of 4.20 ± 0.02 min, theoretical plates *N* = 435,513, and peak symmetry B/A = 0.98 ± 0.02, indicating efficient and reproducible separation across runs.

#### 3.1.4. Calibration Curve

The 4‐ONE calibration curve was constructed using six standard concentrations (0.0032, 0.016, 0.08, 0.4, 2, and 10 ng mL^−1^), each analyzed in triplicate. The peak area data exhibited a strong linear relationship across the entire concentration range, with a correlation coefficient of *R* ^2^ = 0.9993. The slope and intercept of the calibration curve were 742,527 ± 756 and 295,630 ± 293, respectively, with the associated standard deviation confirming the precision of the regression parameters.

**Figure 4:**
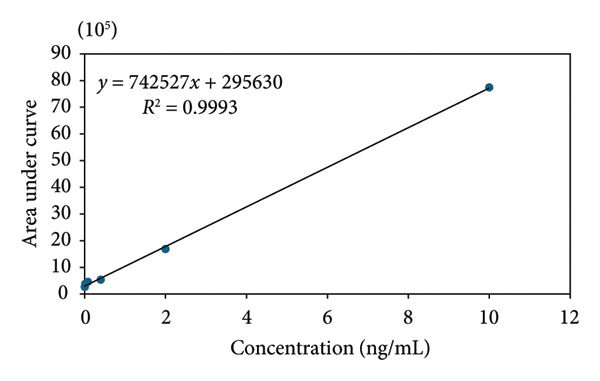
The calibration curve of 4‐oxo‐2‐nonenal (4‐ONE) was constructed using internal and external standards.

Method reproducibility was evaluated by performing the calibration three times consecutively using the same set of standards and three times with freshly prepared standards on consecutive days and weeks (Table 1). Regression analysis consistently yielded stable slope and intercept values while maintaining correlation coefficients above *R* ^2^ = 0.999 in all cases (Figure 4). These results demonstrate that the UPLC‐PDA method provides reliable quantitative performance for 4‐ONE analysis, with reproducible calibration parameters both intraday and interday.

**Table 1:** Reproducibility of calibration curve parameters (slope, intercept, and coefficient of determination) for 4‐oxo‐2‐nonenal (4‐ONE) obtained over three consecutive days within each of 3 weeks.

Day	Week	Slope	±SD	Intercept	±SD	*R* ^2^
1	1	742,300	800	295,500	300	0.9993
2	1	742,600	800	295,700	300	0.9992
3	1	742,450	700	295,550	300	0.9993
1	2	742,800	700	295,830	300	0.9992
2	2	742,500	700	295,600	300	0.9994
3	2	742,700	800	295,650	300	0.9993
1	3	742,400	800	295,520	300	0.9992
2	3	742,530	800	295,600	300	0.9993
3	3	742,464	800	295,760	300	0.9993
Mean average		742,527	800	295,634	300	0.9993

*Note:* Data are expressed as the mean ± SD at a *p* value of *p* > 0.05. Standard error (SE) is 154.4.

#### 3.1.5. System Precision

The UPLC‐PDA chromatogram data for system precision are shown in Table 2. The data for method precision revealed that the % RSD of the concentration from 4 injections (each in duplicate) of the sample solution was below 2% except for 0.2 ng/mL, where the RSD recorded was 2.44% and 2.56%. Other results fall within the specified limits (% RSD < 2.0%) as outlined in the ICH harmonized tripartite guideline of 2005.

**Table 2:** The UPLC chromatogram data for system precision.

Intraday			Interday	
Actual concentration (ng/mL)	Measured concentration (ng/mL)	RSD	Measured concentration (ng/mL)	%RSD
6.0	6.00 ± 0.00	0.08	6.00 ± 0.00	0.00
1.0	1.01 ± 0.01	0.50	1.00 ± 0.01	0.50
0.5	0.50 ± 0.00	0.81	0.50 ± 0.01	0.10
0.2	0.20 ± 0.50	2.44	0.5 1 ± 0.00	2.56

*Note:* Values are means ± standard deviation of three independent replicates performed at three different occasions.

#### 3.1.6. Recovery

The method’s accuracy was evaluated through recovery experiments at four concentration levels. The concentrations tested were 1, 0.2, 0.4, and 0.03 ng/mL, and the corresponding measured values were found to be 0.97, 0.21, 0.39, and 0.03 ng/mL, as shown in Table 3 and the corresponding chromatograms in Figure 5. This demonstrates the sensitivity of the developed method.

**Table 3:** Recovery of 4‐oxo‐2‐nonenal at four different concentrations^∗^.

Concentration (ng/mL)	Calculated spiked concentration (ng/mL)^∗^	±SD	RSD (%)	Recovery (%)
1.00	0.97	0.02	1.75	97.16
0.20	0.21	0.00	0.85	105.90
0.40	0.39	0.01	1.26	99.55
0.03	0.03	0.00	1.07	101.92

*Note:* Values are means ± standard deviation of three independent replicates performed at three different occasions.

^∗^Four different concentrations were used (1, 0.20, 0.40, and 0.03 ng/mL).

Figure 5Chromatogram of 4‐oxo‐2‐nonenal for recovery study at (a) 0.4 ng/mL; (b) 0.2 ng/mL; and (c) 0.03 ng/mL.

**Figure:**
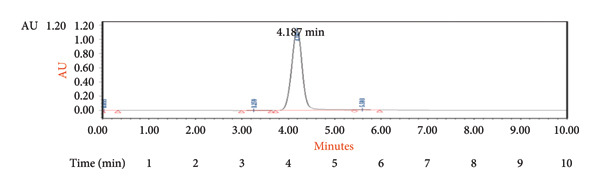
(a)

**Figure:**
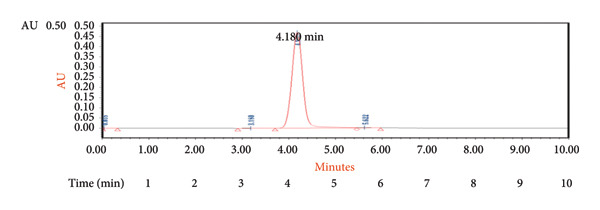
(b)

**Figure:**
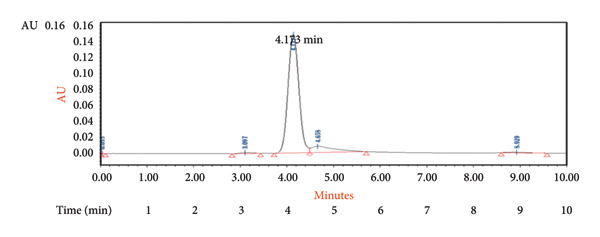
(c)

#### 3.1.7. RT

**Figure 6:**
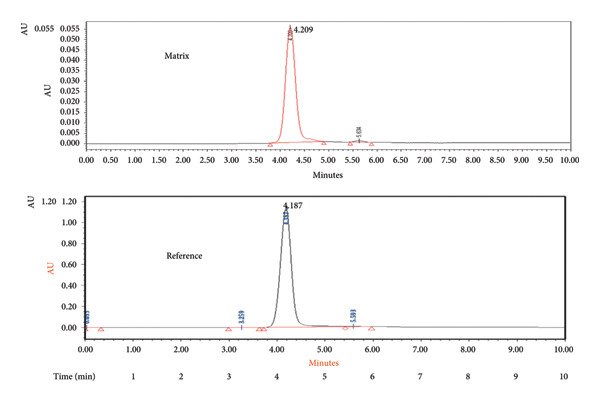
The mean retention time (RT) and reference retention time (RT) for the matrix were estimated as 4.209 and 4.187 min, respectively.

As stated by Kumari et al. [18], estimating RT is an aspect of method validation. First, it helps identify the peaks of the analyte within a sample or matrix and their corresponding reference in calibration standards. Additionally, any variation in RT can indicate errors during the extraction process. In chromatography, it is generally assumed that the analyte present in the matrix should exhibit a RT equal to that of the calibration standard with an acceptable level of deviation. The mean RT for matrix and reference RT were estimated as 4.2 and 4.18 min, respectively. The absolute difference in RT was calculated as 0.02 min (Figure 6).

#### 3.1.8. Robustness

**Figure 7:**
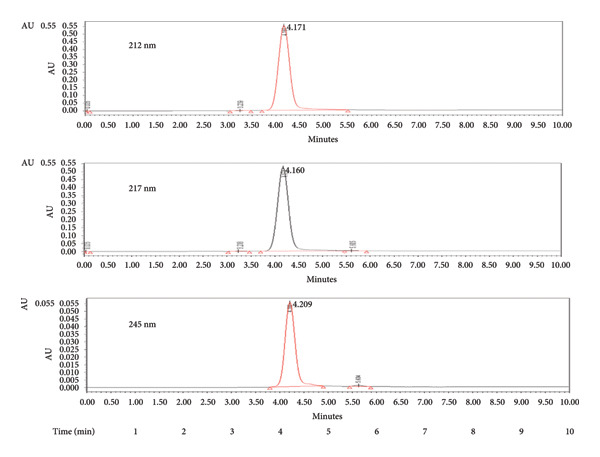
Chromatograph of 4‐oxo‐2‐nonenal at different detection wavelengths (212, 217, and 245 nm).

The data revealed that the % RSD for decrease and increase in flow rate for 4‐ONE was 0.01 and 0.00, respectively, within the specified limits (% RSD NMT 2.0%). The % RSD for 75:25 and 80:20 mobile phase ratios were 0.01 and 0.02, respectively, within the specified limits (% RSD NMT 2.0%). % RSD for decrease and increase in wavelength were 0.07 and 0.70, respectively, within the specified limits (% RSD NMT 2.0%). From the above study, it can be established that the flow rate, mobile phase ratio, and wavelength are robust to allowable variations. The data are presented in Table 4 and Figure 7.

**Table 4:** Robustness of the UPLC‐PDA method for 4‐oxo‐2‐nonenal.

Parameter	Condition	Mean peak area	±SD (peak area)	%RSD (peak area)	Mean RT (min)	±SD (RT)	%RSD (RT)
Flow rate (mL/min)	0.02	53,238	60	0.01	4.21	0.00	0.24
	0.05	53,280	50	0.00	4.19	0.00	0.22

Wavelength (nm)	212	53,273	400	0.07	4.20	0.00	0.48
	230	53,272	20	0.00	4.18	0.00	0.23
	245	53,363	3700	0.70	4.20	0.00	0.47

Mobile phase ratio (A:B)	80:20	53,296	90	0.02	4.21	0.00	0.21

Mobile phase ratio (A:B)	75:25	53,316	40	0.01	4.22	0.00	0.24

*Note:* Values represent the mean of triplicate injections performed under each robustness condition.

## 4. Conclusion

This study has effectively developed a UPLC‐PDA technique for detecting and quantifying 4‐ONE in cooked meat. The method has shown linearity, precision, accuracy, and robustness, making it suitable for analysis in food labs. By optimizing parameters such as the flow rate, injection volume, and mobile phase composition, the method successfully achieved well‐defined peaks, ensuring the accurate measurement of 4‐ONE. The validation results met the required criteria, confirming the reliability and suitability of the process for analyzing 4‐ONE in meat samples. The successful development of this method contributes to our understanding of oxidation products in cooked foods, while also helping to ensure food safety and quality. Future research can explore the use of this method to analyze compounds in various types of food, providing valuable insights into the complex chemistry involved in cooking.

## Ethics Statement

The authors declare that all ethical guidelines were followed in the conduct of this study. Where applicable, informed consent and institutional approvals were obtained.

## Disclosure

The findings of this manuscript have been presented as a poster in the “Abstract of 22^nd^ World Congress of Food Science and Technology (IUFoST 2024).”

## Conflicts of Interest

The authors declare no conflicts of interest.

## Funding

The authors received no specific funding for this work.

## Data Availability

The data that support the findings of this study are available from the corresponding author upon reasonable request.
